# Laryngomalacia Presenting as Recurrent Croup in an Infant

**DOI:** 10.1155/2013/649203

**Published:** 2013-01-27

**Authors:** Osama Elbuluk, Travis Shiba, Nina L. Shapiro

**Affiliations:** Department of Head and Neck Surgery, David Geffen School of Medicine, UCLA, Room 62-158 CHS, P.O. Box 951624, 10833 Le Conte Avenue, Los Angeles, CA 90095, USA

## Abstract

Laryngomalacia is a common disease of infancy which can present with atypical symptoms and at an atypical age, causing the diagnosis to often be overlooked. We report a case of a male patient who was diagnosed with laryngomalacia at the age of three months. The patient's inspiratory stridor resolved within a year, but he went on to develop atypical croup. The patient was later diagnosed with severe laryngomalacia which complicated his “croup-like” symptoms. He subsequently underwent supraglottoplasty with complete resolution of symptoms.

## 1. Introduction

Laryngomalacia is defined as the collapse of supraglottic structures during inspiration [1]. It is the most common cause of inspiratory stridor in infants and affects 45–75% of all infants with congenital stridor [2]. Inspiratory stridor is the hallmark symptom, but occasionally more severe cases can be associated with feeding difficulties such as choking and regurgitation as well as intermittent episodes of hypoxia [3]. In the majority of patients, symptoms will peak between six and eight months and resolve between twelve and twenty-four months [2]. At the time of presentation, 40% of patients will have mild disease with inconsequential stridor, 40% will have moderate disease with feeding complications, and 20% will have severe disease [4]. Most mild cases can be routinely monitored by a pediatrician, with referral to an otolaryngologist if symptoms worsen or persist. Patients with severe disease may experience less common symptoms such as tachypnea, suprasternal or substernal retractions, pectus excavatum, and obstructive sleep apnea [4]. These patients often require surgical management with supraglottoplasty as the mainstay of therapy. Depending on outcomes measured, surgical intervention has proven to be successful in up to 94% of cases [5].

## 2. Case Report

A 3-month-old boy presented with noisy breathing at birth which was reportedly noisier while in the supine position. The patient's history was significant for delivery at 34 weeks secondary to premature rupture of membranes. After receiving a 5-day postnatal course of prophylactic antibiotics, as well as steroids for lung development, he was discharged to home. At home, he had been feeding well and had no history of apnea or cyanosis.

After being seen by his pediatrician at the age of 3 months for intermittent inspiratory stridor, the patient was referred to our pediatric otolaryngology clinic for further evaluation. On examination, the patient had mild inspiratory stridor and did not appear to be in any distress. There were no signs of retractions, and both his otologic and nasal exam were normal. He had no retrognathia or craniofacial dysmorphism. Flexible fiberoptic nasopharyngolaryngoscopy revealed mild-to-moderate laryngomalacia with intact, symmetric vocal cord mobility, and no evidence of a glottic or supraglottic mass. The severity of laryngomalacia was defined by the extent of disease on laryngoscopy (omega-shaped epiglottis, foreshortened aryepiglottic folds, with ability to visualize the true vocal cords) as well as frequency and severity of symptoms. At that time, the patient was sent home with return instructions.

**Figure 1:**
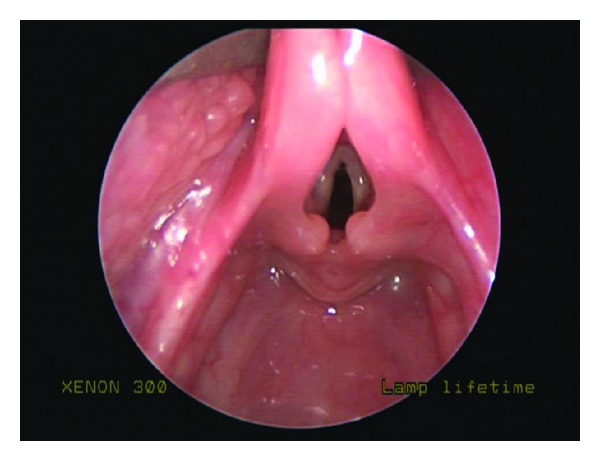
Laryngomalacia prior to surgical intervention. Redundant supraarytenoid tissue can be seen obstructing the glottis.

**Figure 2:**
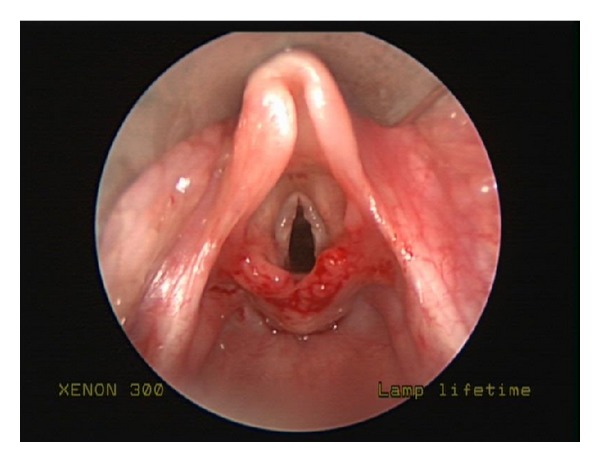
Status after supraglottoplasty.

The patient's stridor persisted until the age of 11 months, but at the age of one year his breathing began to improve. However, at that time, the patient developed recurrent croup. Croup was diagnosed by the patient's pediatrician based on his “bark-like” cough which differed from the inspiratory stridor seen with his laryngomalacia. Approximately every two months thereafter, the patient required treatment with steroids and epinephrine nebulizers for treatment of his croup. At the age of 2 years, the patient was referred back to our clinic for reevaluation. At that time, he was noted to have faint inspiratory stridor at rest, as well as the parental history of recurrent croup for over one year. It was decided at that point to proceed with operative evaluation. The patient underwent suspension microlaryngoscopy and was found to have severe elongation of the epiglottis with redundancy of the aryepiglottic folds as well as elongation of the arytenoids with redundant arytenoid mucosa and foreshortened aryepiglottic folds (Figure 1). The subglottis and trachea were normal, with no evidence of subglottic stenosis or edema. The patient underwent supraglottoplasty, whereby the aryepiglottic folds were divided bilaterally and the redundant arytenoid mucosa was trimmed with microlaryngeal scissors (Figure 2). The patient tolerated the procedure well with resolution of symptoms on the night postoperatively. The patient remained asymptomatic when seen two weeks later at postoperative follow-up. He remains asymptomatic at 2 months after surgery. 

## 3. Discussion

Laryngomalacia is a common pathology seen in early infancy. The vast majority of patients experience a benign disease course with the resolution of symptoms within the first twelve to eighteen months, without the need for operative intervention [2]. It is believed that the laryngomalacia resolves secondary to the maturation of the central nervous system [6]. Studies have shown that an alteration of the laryngeal adductor reflex can result in inappropriate glottal closure and this may play a role in the etiology of laryngomalacia in patients with moderate-to-severe disease [6]. Our report aims to draw attention to the notion that in the setting of a concomitant airway disorder, such as recurrent croup, the severity of laryngomalacia may be masked. This patient had a prolonged presence of laryngomalacia, necessitating surgical intervention after infancy.

As previously mentioned, it is fairly rare for a patient's laryngomalacia to remain symptomatic well past infancy. 70% of patients with mild disease endure an uneventful disease course, while 30% progress to moderate disease [4]. Similarly, 72% of patients with moderate disease have resolution of symptoms with lifestyle modifications, while 28% progress to severe disease [4]. In our case, the patient presented with mild-to-moderate disease but progressed to severe disease which eventually required surgical correction. Intervening at this point in the disease progression was vital, as the laryngeal cartilage was still immature and pliable. The older the patient, the more technically difficult the operation and the less likely one is to see the complete resolution of gross disease on laryngoscopy. However, this particular patient had no clinical evidence of gastroesophageal reflux disease or additional comorbidities, making the need for future revision supraglottoplasty very unlikely.

In addition, this case shows how easily laryngomalacia can be disguised in its clinical presentation. Initially, it is frequently misdiagnosed, with diagnoses such as bronchiolitis, asthma, tracheomalacia, and reactive airway disease often preceding the appropriate diagnosis [4]. Zoumalan et al. found that in patients who were diagnosed by a non-otolaryngologist, 30% were receiving treatment for an incorrect diagnosis, the most common being tracheomalacia [7]. Our patient, despite having a previous diagnosis of mild-to-moderate laryngomalacia, presented to his pediatrician with what was diagnosed as recurrent croup. In retrospect, it was not that croup was misdiagnosed, but rather croup symptoms were magnified by the presence of baseline laryngomalacia. In this case, we do not believe laryngomalacia caused croup but rather hypothesize that the two airway lesions acted in synergy to produce more severe, persistent disease. Cooper et al. performed a review of patients who were sent to a tertiary pediatric referral center for further investigation of atypical croup, where the child either had recurrent episodes or was of abnormal age for contracting croup [8]. Of the 80 patients studied, only 31 patients had positive airway findings, with only three demonstrating pathology consistent with laryngomalacia [8]. Patients presenting with atypical croup or delayed resolution of symptoms should be evaluated with upper airway endoscopy to search for reversible causes, which, although rare, can include severe laryngomalacia.
